# Sensitive detection of human papillomavirus type 16 E7-specific T cells by ELISPOT after multiple *in vitro *stimulations of CD8^+ ^T cells with peptide-pulsed autologous dendritic cells

**DOI:** 10.1186/1476-4598-5-49

**Published:** 2006-10-26

**Authors:** Nathalie Cools, Peter Ponsaerts, Marc Lenjou, Griet Nijs, Dirk R Van Bockstaele, Viggo FI Van Tendeloo, Zwi N Berneman

**Affiliations:** 1 Laboratory of Experimental Hematology, Antwerp University, Faculty of Medicine, Antwerp University Hospital, Belgium

## Abstract

**Background:**

Cervical cancer is the second most common gynecological cancer amongst women world-wide. Despite optimized protocols, standard treatments still face several disadvantages. Therefore, research aims at the development of immune-based strategies using tumor antigen-loaded dendritic cells for the induction of cellular anti-tumor immunity.

**Results:**

In this study, we used dendritic cells loaded with the HLA-A2-restricted HPV type 16 E7_11–20 _peptide in order to induce an *in vitro *CD8^+ ^T cell response. For this purpose, peptide-pulsed dendritic cells were co-cultured with autologous CD8^+ ^T cells. After 5 weekly stimulations with peptide-pulsed mature dendritic cells, cultured T cells were analyzed for antigen specificity by an IFN-γ ELISPOT assay. Using this ELISPOT assay, we were able to detect E7-specific IFN-γ-secreting CD8^+ ^T cells in 5/5 healthy donors.

**Conclusion:**

We show that peptide-pulsed mature dendritic cells are able to stimulate a HPV type 16 E7 peptide-specific immune response *in vitro*. These experiments describe an efficient culture protocol for antigen-specific T cells for use in pre-clinical vaccination research and confirm the need for sensitive T cell assays for detection of tumor-specific immune responses *in vitro*.

## Background

Cervical cancer contributes to approximately 12% of the global cancer burden in women and represents the second most frequent gynecological malignancy in the world [1,2]. At an early stage, cervical cancer is treated by means of surgery and radiotherapy [3]. In a more advanced stage, one uses a combination therapy of cisplatinum-containing chemotherapy and radiotherapy [4]. Despite technological progress in conventional treatment modalities, more than 35% of patients develop a metastasizing malignancy with poor results after treatment. An additional disadvantage of radio- and chemotherapy is a pronounced and long-lasting negative effect on the immune system. For these reasons, current research aims at the development of new and more efficient strategies. In this context, dendritic cells (DC) loaded with tumor antigens for activation and/or expansion of tumor-specific T cells are currently the subject of intensive research in the field of cellular immunotherapy [5-7].

One of the major risk factors for the development of cervical cancer is infection with human papillomavirus (HPV). More than 20 oncogenic HPV genotypes have been characterized, while HPV type 16 (HPV-16) and type 18 (HPV-18) are the most prevalent in cervical cancer [8]. In HPV-16 positive tumors, the E7 oncoprotein is constitutively expressed in cervical tumor cells [9] and is responsible for transformation of these cells [10]. Moreover, HPV-16 E7-specific cytotoxic T lymphocytes (CTL) have been demonstrated in the peripheral blood, the lymph nodes and the tumor tissue of HPV-16-positive cervical carcinoma patients [11,12]. An effective HPV-specific cellular immune response can be generated after active immunization [13-16]. For these reasons, the HPV-16 E7 protein is a target of choice for the development of a specific immune therapy directed against cervical cancer.

Stimulation of the immune system against specific tumor antigens might become a suitable secondary (or even primary) therapy to treat cancer. Because tumor cells do not efficiently function as antigen-presenting cells (APC) for the activation of CTL, a vigorous immune response is generally absent or defective. To overcome this problem, transfer of tumor antigens from tumor cells to professional APC, like DC, might be a valuable strategy [17].

DC are the most efficient antigen-capturing and -presenting cells of the immune system and are potent inducers of (primary) immune responses directed against tumors and viral antigens [18]. In their immature state, DC are skilled in antigen uptake by means of endocytosis and phagocytosis. After uptake, antigens are processed by DC to peptide fragments which are bound to major histocompatibility complex (MHC) class I and II molecules. After transport to the plasma membrane, these peptide-MHC complexes can be recognized by a T cell receptor (TCR) with high specificity for the antigenic peptide-MHC complex. If antigen uptake and presentation is associated with danger signals, provided by microbial components such as lipopolysaccharide (LPS) [19], DC are activated and an effective immune response can be induced.

In this study, we used *in vitro *cultured mature DC loaded with an HLA-A*0201-restricted HPV-16 E7 peptide for the *in vitro *activation of antigen-specific IFN-γ-producing CD8^+ ^T cells. After several rounds of *in vitro *stimulation of CD8^+ ^T cells using peptide-pulsed mature DC, the presence of HPV-16 E7 peptide-specific T cells was monitored using an IFN-γ enzyme-linked immunospot (ELISPOT) assay.

## Results

### Phenotypic analysis of mature monocyte-derived DC for T cell stimulation

**Figure 1:**
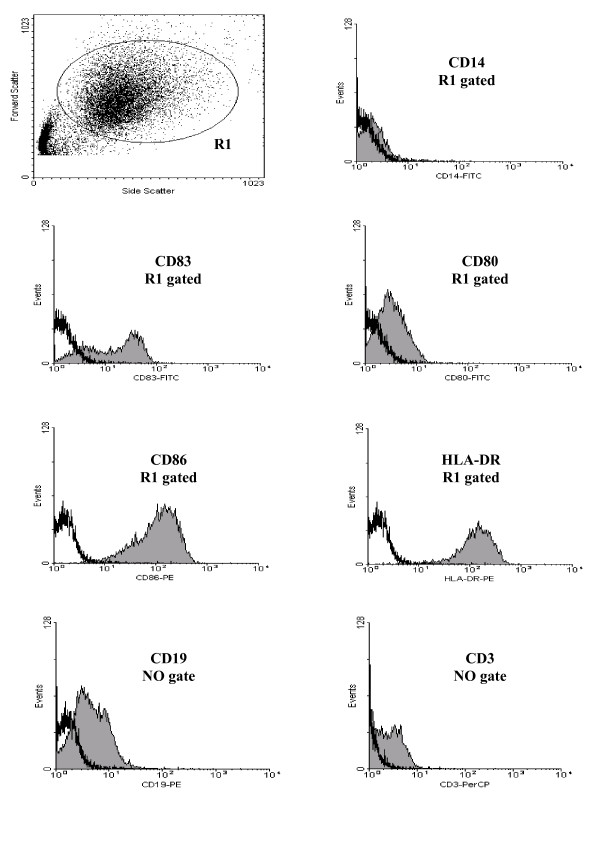
**Immunophenotypic analysis of dendritic cells**. Plastic adherent monocytes were cultured in the presence of GM-CSF and IL-4 for 6 days, followed by activation with a cytokine cocktail consisting of IL-1, IL-6, TNF-α and PGE_2 _for 24 hours (filled histogram). Analysis of typical DC membrane markers (CD80, 83, 86 and HLA-DR) and the monocyte marker CD14 was determined on R1 as shown in the forward vs side scatter dot plot, CD3 and CD19 on the ungated population. An isotype control is represented by the bold line.

Mature DC were differentiated starting from adherent monocytes as described in the Methods section [20]. Cultured DC were phenotypically characterized for the expression of typical DC membrane markers (HLA-DR, CD80, CD83 and CD86) by flow cytometry (Figure 1). Analysis was restricted to DC by gating based on characteristic forward and side scatter clustering (Figure 1: R1 gate in forward vs side scatter dot plot). The mature DC population was negative for the monocyte marker CD14 and positive for costimulatory molecules HLA-DR, CD80 and CD86 and the adhesion receptor CD83. Moreover, the final DC populations displayed minimal contamination of T and B cells as analyzed by CD3 and CD19 expression on the cultured DC population (Figure 1: no gate).

### Peptide-pulsed DC efficiently present a MHC class I-restricted E7 peptide to CD8^+ ^T cells

**Figure 2:**
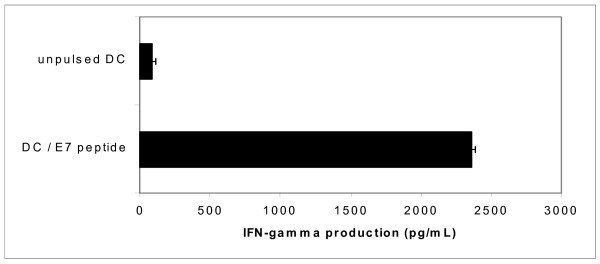
**IFN-γ production by an HPV type 16 E7 peptide-specific HLA-A2^+ ^T cell clone shows that peptide-pulsed mature DC efficiently bind and present the E7 peptide to T cells**. HPV-16 E7-peptide-pulsed or non-pulsed mature DC were used as stimulator cells. Mature DC were co-incubated with a HPV-16 E7-specific CD8^+ ^CTL clone. After 6 h, IFN-γ released in the co-culture was determined by IFN-γ ELISA. The T cell clone produced 2364 ± 28 pg/mL IFN-γ upon E7 peptide-pulsed mature DC stimulation compared to 83 ± 21 pg/mL IFN-γ upon control stimulation (p-value = 0.001).

First, we evaluated if our cultured mature DC population was able to present the YMLDLQPETT peptide to CD8^+ ^T cells in a MHC class I-dependent way. For this, E7 peptide-pulsed mature DC (2.10^4 ^cells) as well as non-pulsed mature DC (2.10^4 ^cells) were co-cultured with an HLA-A*0201^+ ^HPV-16 E7 YMLDLQPETT peptide-specific T cell clone (2.10^5 ^cells) for 6 hours in 96-round bottom wells. As showed by the production of IFN-γ by the T cell clone, E7-peptide-pulsed mature DC were able to stimulate these antigen-specific T cells (Figure 2, p = 0.001).

### Detection of immune responses against HPV-16 immunodominant E7 peptide

Previously, we reported a DC-based T cell activation protocol for influenza matrix protein M1 peptide-specific T cells [21]. We wanted to evaluate whether this T cell activation protocol was equally efficient in stimulating HPV-16 E7-specific autologous CD8^+ ^T cells. However, after a 7 day *in vitro *stimulation period of peripheral blood mononuclear cells (PBMC) with HPV-16 E7 peptide-pulsed mature DC, we were not able to detect an HPV-16 E7-specific immune response (data not shown). We therefore added to the protocol 4 additional weekly stimulations with HPV-16 E7-peptide-pulsed mature DC and implemented an IFN-γ ELISPOT technique for detection of antigen-specific IFN-γ-secreting T cells.

**Figure 3:**
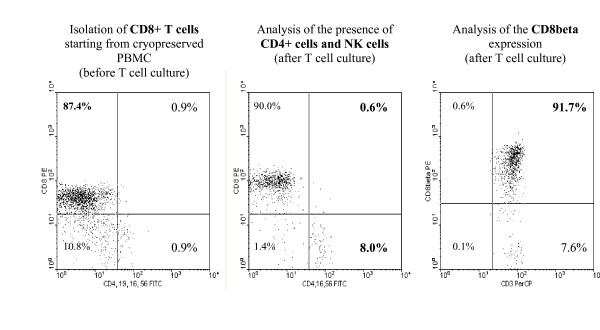
**Representative example of the phenotypic analysis of the effector T cell population**. CD8^+ ^T cells were isolated by magnetic separation using CD8 microbeads. Dot plots were gated on the lymphocyte population based on forward and side scatter properties. The indicated percentages are within the lymphocyte population.

DC were differentiated starting from monocytes and matured with a maturation cocktail consisting of IL-1, IL-6, TNF-α and PGE_2 _[22]. Co-cultures were set up in multiple 96-round-bottomed wells. In each well, 15.10^3 ^HPV-16 E7-peptide-pulsed DC were cocultured together with 15.10^4 ^autologous CD8^+ ^T cells, isolated using magnetic bead selection from cryopreserved autologous PBMC (Figure 3). Growth factors (IL-2 and IL-7) were added weekly to the T cell medium starting from week 2, together with a total of 4 additional weekly stimulations with cryopreserved HPV-16 E7 peptide-pulsed DC. We evaluated the presence of a detectable immune response by determining the presence of IFN-γ-producing cells after restimulation with control or E7 peptide-pulsed targets by means of an IFN-γ ELISPOT. For this analysis we used non-pulsed K562-A*0201 cells as control targets and K562-A*0201 cells [23] pulsed with the HPV-16 E7 peptide as HPV-16 E7 peptide-specific target cells in a 10:1 effector:target cell ratio. Furthermore, the effector T cell population was phenotypically evaluated in order to address the nature of the effector T cell population with certainty. Figure 3 shows that the majority of effector cells exists of CD8β^+ ^T cells. CD8β is a specific marker for cytotoxic T cells as it is not expressed by CD8α^+ ^NK cells or CD4^+ ^T helper cells.

**Figure 4:**
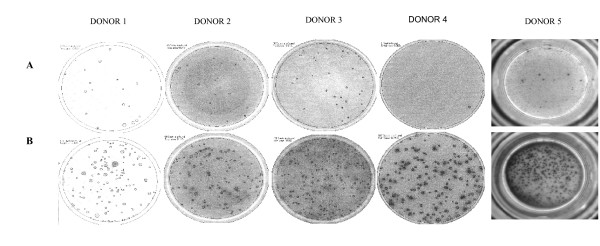
**Representative presentation for each donor of a positive well (B) compared to a control well (A) in the IFN-γ ELISPOT plate**. CD8^+ ^T cells stimulated multiple times with HPV-16 E7 peptide-pulsed mature DC were analyzed for antigen-specific IFN-γ secretion by means of IFN-γ ELISPOT. For this analysis cultured CD8^+ ^T cells were stimulated with HPV-16 E7 peptide-pulsed K562-HLA-A*0201 cells (B), while non-pulsed K562-A*0201 cells were used as control cells (A).

Using the IFN-γ ELISPOT assay in several individual experiments (n = 48) tested per blood donor, a significant difference was detected between the number of spot-forming CD8^+ ^T cells (SFC: spot-forming cells) in response to HPV-16 E7-peptide-pulsed target cells as compared to control targets (Table 1). Figure 4 shows a representative picture of a positive well per blood donor as compared to a control well on the ELISPOT plate. These results confirm that HPV-16 E7-specific T cells can be sufficiently activated and expanded by multiple stimulations with peptide-pulsed mature DC to a detectable level for analysis with an IFN-γ ELISPOT assay.

**Table 1:** Analysis of HPV-16 E7 antigen-specific IFN-γ secretion by means of the IFN-γ ELISPOT assay

**DONOR**	**Fifth stimulation Positive cultures/total cultures**	**Percentage of antigen-specific spot forming cells (SFC) in positive wells**
1	2/48	0.03–0.08 [0.06]
2	3/48	0.05–0.06 [0.05]
3	6/48	0.02–0.04 [0.03]
4	7/48	0.03–0.18 [0.08]
5	2/2	0.12–0.14 [0.13]

Responses were considered positive if a minimum of 15 SFC were counted after the control had been subtracted and if the ratio of SFC in positive wells to that in control wells was > 3. The range of the percentage of antigen-specific SCF is also given, with the mean percentage between square brackets, calculated as the ratio between the number of spots in a positive well and the total number of T cells in that well.

## Discussion

Adequate immune monitoring techniques are of utmost importance to validate phase I/II clinical trials designed to test T cell-based vaccines for HPV-related malignancies. Such endpoints include the measurement of HPV-specific CTL- and T helper-mediated immune responses. Routinely used techniques such as ELISA and RT-PCR, do not provide information regarding the frequencies of cytokine-producing cells, in contrast to sensitive methods such as ELISPOT.

In this study, we show efficient detection of HPV-16 E7 peptide-specific IFN-γ-producing autologous T cells after multiple stimulations with HPV-16 E7-peptide-pulsed mature DC using an IFN-γ ELISPOT assay (Figure 4 and Table 1). In contrast, we could not detect HPV-16 E7-specific T cells with a previously used IFN-γ ELISA nor with a LDH release cytotoxicity assay (data not shown). This laborious detection of a HPV-16 E7-specific immune response in healthy donors could be due to multiple factors. The weak sensitivity of bulk assays such as ELISA and LDH release lysis assay can be an influencing factor. The sensitivity of ELISPOT is indeed higher than that of an ELISA. It is necessary to have at least 400 cells in an ELISA system to produce 1 pg of cytokine. In contrast, in an ELISPOT assay, one assumes that 1 IFN-γ-secreting cell is detectable among 5.10^5 ^cells [24]. Another important limiting factor is the low precursor frequency of HPV-specific CTL in healthy volunteers [8]. For example, one stimulation with peptide-pulsed mature DC does probably not induce sufficient expansion of HPV-16 E7-specific T cells. Therefore, the T cell stimulation protocol included a total of 5 weekly stimulations with HPV-16 E7-peptide-pulsed mature DC. Moreover, our results are in agreement with previous studies demonstrating the induction of an HPV-16 E7-specific immune response in healthy volunteers after multiple *in vitro *stimulations with autologous antigen-loaded mature DC [14,15]. However, in contrast to Schoell et al. [14], we have used human AB serum instead of fetal calf serum in our experiments, in order to pave the way to future clinical application of this culture protocol. Moreover, the nature of the effector T cell population was more critically evaluated. By using the CD8β marker, we ascertained that the majority of the IFN-γ-producing effector population consists of CD8β^+ ^T cells. Also, we excluded aspecific IFN-γ production by CD4^+ ^T helper cells or NK cells by subtracting control values for our estimation of effector CD8^+ ^T cell frequencies.

## Conclusion

Taken together, we showed that peptide-pulsed mature DC are able to induce a cellular response against HPV-16 in 5/5 healthy volunteers, as detected by E7-specific IFN-γ producing CD8^+ ^T cells in an ELISPOT analysis. The importance of this study is that it presents an efficient DC-based culture protocol for tumor antigen-specific T cells in pre-clinical vaccination studies. Moreover, our observations confirm the need for sensitive T cell detection methods, such as ELISPOT, for the detection of tumor specific immune responses *in vitro*.

## Methods

### Cell lines

K562-HLA-A*0201 cells were kindly provided by Dr. C. Britten (Johannnes-Gutenberg University, Mainz, Germany) [23]. Cells were cultured in complete medium consisting of Iscove's modified Dulbecco's medium supplemented with L-glutamine (IMDM; Invitrogen, Paisley, UK), gentamicin (10 μg/mL, Invitrogen), amphotericin B (1.25 μg/mL Fungizone; Invitrogen) and 10 % heat inactivated fetal calf serum (FCS; Sera Lab, Sussex, UK) and the neomycin analogue G418 (0.5 mg/mL, Invitrogen). Cells were maintained in logarithmic phase growth at 37°C in a humidified atmosphere supplemented with 5% CO_2_.

### HPV type 16 E7 peptide-specific T cell clone

The CD8^+ ^D4 T cell clone recognizing the immunodominant HLA-A*0201-restricted HPV-16 E7 epitope (YMLDLQPETT) was a kind gift of Dr. S. Youde (University of Wales College of Medicine, Cardiff, UK). Cloned T cells (1.10^6^) were expanded to large numbers in tissue culture flasks containing 50 mL IMDM supplemented with 10% human (h)AB serum (Sigma, Cambridge, UK) with 20.10^6 ^fresh irradiated (30 Gy) allogeneic peripheral blood mononuclear cells (PBMC) from 2 donors, phytohemagglutin A (PHA) (1 μg/mL, Sigma) and interleukin (IL)-2 (200 U/mL, Biosource, Camarillo CA, USA). Fresh medium and IL-2 were added on day 5. On day 7, the T cells were transferred to a 24-well plate at 2.10^6 ^cells/well and cultured in the presence of 100 U/mL IL-2 for up to 14 days.

### Source of primary cells

Peripheral blood mononuclear cells (PBMC) were obtained from hemochromatosis patients after informed consent or from buffy coats provided by the Antwerp Blood Transfusion Center (Red Cross). Mononuclear cells were isolated by Ficoll-Hypaque gradient separation (LSM, ICN Biomedicals, Costa Mesa, CA, USA). Monocytes were directly isolated and used for DC culture, as described below.

### In vitro culture of DC

Immature monocyte-derived DC were cultured from monocytes as described by Romani *et al*. [20] with minor modifications. Briefly, PBMC were allowed to adhere in serum-free AIM-V medium (Invitrogen) for 2 h at 37°C. The non-adherent cell fraction was cryopreserved in a solution consisting of 90% FCS and 10% DMSO and stored at -80°C until later use in DC/T cell co-cultures,. Adherent monocytes were further cultured for 6 days in IMDM supplemented with 5% hAB serum (Sigma). One hundred ng/mL granulocyte-macrophage colony-stimulating factor (GM-CSF; Leucomax, Novartis Pharma, Basel, Switzerland) and 1000 U/mL IL-4 (R&D Systems, Minneapolis, MN, USA) were added to the cultures. Maturation of immature DC was induced on day 6 by adding a maturation cocktail consisting of 100 U/mL IL-1 (Biosource Europe, Nivelles, Belgium), 500 U/mL IL-6 (Biosource), 2.5 ng/mL tumor necrosis factor (TNF)-α (Roche Molecular Biochemicals, Mannheim, Germany) and 10^-7 ^M prostaglandin E_2 _(PGE_2_) (Sigma, St. Louis, MO, USA), as previously described by Jonuleit *et al *[22]. Mature DC were either directly used in DC/T cell co-culture or cryopreserved for further restimulations as previously described [21]. Briefly, cells were resuspended in a solution consisting of 90% FCS and 10% DMSO and were stored at -80°C until use.

### Flow cytometry

Screening of blood donors for HLA-A*0201-positivity was performed on whole blood by incubation with the supernatant of the BB7-2 hybridoma (anti-HLA-A*0201; ATCC, Manassas, VA, USA), followed by staining with fluorescein (FITC)-conjugated rabbit anti-mouse immunoglobulins (DAKO Cytomation, Heverlee, Belgium). For phenotyping of cultured DC and T cell populations, the following murine anti-human monoclonal antibodies were used for direct immunofluorescence staining: CD80-FITC, CD83-phycoerythrin (PE) (Santa Cruz Biotechnology, Santa Cruz, CA, USA), CD86-PE, CD3-peridinin chlorophyll-a protein (PerCP), CD14-PE, CD19-FITC, HLA-DR-PE, CD4-FITC, CD16-FITC, CD56-FITC, CD8-PE and CD8β-PE. Non-reactive isotype-matched antibodies were used as controls. All antibodies were purchased from Becton Dickinson (Erembodegem, Belgium), unless stated otherwise. Cells were stained with antibody for 15 min at 4°C, washed and surface labelling of living cells was analyzed by flow cytometry on a FACScan (Becton Dickinson).

### Peptide pulsing of DC and K562-HLA-A*0201 cells

An HPV-16 HLA-A*0201-restricted E7 protein-specific peptide (E7; amino acids (aa) 11–20, YMLDLQPETT) was used for activation or for detection of HPV-16 E7 peptide-specific T cells, when pulsed on DC or on K562-HLA-A*0201 cells respectively. The peptides (>95% pure) were purchased from Eurogentec (Seraing, Belgium) and were dissolved in 100% DMSO to 10 mg/mL, further diluted to 1 mg/mL in serum-free IMDM and stored in aliquots at -80°C. The peptides were used at a final concentration of 20 μg/mL. DC or K562-HLA-A*0201 cells were washed once with IMDM and subsequently incubated (1.10^6 ^cells/mL) for 2 h at room temperature in 5-mL polystyrene tubes with 20 μg/mL peptide in serum-free IMDM supplemented with 2.5 μg/mL β_2_-microglobulin (Sigma). Peptide-pulsed DC and K562-HLA-A*0201 cells were used, respectively, as stimulators for T cells in DC/T cell co-cultures and as antigen-specific stimulator cells in an IFN-γ ELISPOT assay.

### CD8^+ ^T cell isolation

For DC/T cell co-culture experiments, frozen non-adherent PBMC were thawed and CD8^+ ^T cells were isolated by magnetic separation using CD8 microbeads (Miltenyi Biotec, Bergisch Gladbach, Germany) according to manufacturer's instructions. Routinely, 10–20.10^6 ^CD8^+ ^T cells were obtained starting from 100.10^6 ^cryopreserved non-adherent cells with purity levels ≥ 85%.

### Induction of MHC class I-restricted HPV-16 E7-peptide-specific T cells

HPV-16 E7 peptide-pulsed mature monocyte-derived DC were used for stimulation of autologous CD8^+ ^T cells. Multiple co-cultures of 15.10^3 ^peptide-pulsed DC with 15.10^4 ^purified CD8^+ ^T cells were initiated in AIM-V medium supplemented with 5% hAB serum in 96-round bottom wells (Costar, Corning, NY, USA). After 7 days of co-culture, T cells were restimulated with 15.10^3 ^cryopreserved peptide-pulsed mature DC. At this point, additional cytokines were added to the co-cultures: 5 ng/mL IL-7 (Biosource) and 10 U/mL IL-2 (Biosource). Restimulation of cultured T cells was done weekly. After 5 stimulations, T cells were analyzed for antigen specificity in an IFN-γ ELISPOT assay.

### IFN-γ release assays

HPV-16 E7 peptide-pulsed mature DC were used as stimulator cells for the E7 peptide-specific D4 CTL, while non-pulsed mature DC were used as control cells. CTL (2.10^5 ^cells) were co-incubated with stimulator cells (2.10^4 ^cells) in 96-round bottom plates for 6 hours at 37°C in a total volume of 200 μL IMDM supplemented with 5% hAB serum. Triplicate supernatant samples from these co-cultures were tested for IFN-γ secretion by an IFN-γ enzyme-linked immunosorbant assay (ELISA) (Biosource). Measurements are presented as pg/mL released IFN-γ by 2.10^5 ^CTL per 6 hours. Results are expressed as the mean ± standard deviation. Comparisons were validated using Student's *t *test.

### IFN-γ ELISPOT assay

K562-HLA-A*0201 cells, pulsed with HPV-16 E7 protein-specific peptide, were used as stimulators of cultured CD8^+ ^T cells in an IFN-γ ELISPOT assay, while non-pulsed K562-HLA-A*0201 cells were used as control cells. Stimulator and responder cells were washed and resuspended in AIM-V supplemented with 5% hAB serum. Then, responder T cells (5.10^4 ^cells) were co-incubated with stimulator cells (1.10^4 ^cells) overnight at 37°C in anti-IFN-γ antibody coated 96-well PVDF-plates (Millipore, Bedford, MA, USA) in a total volume of 100 μL. These plates were further analyzed for IFN-γ secretion by IFN-γ ELISPOT staining technique (Diaclone, Amsterdam, The Netherlands) according to manufacturer's instructions. Frequencies of antigen-specific IFN-γ-secreting cells were calculated based on the number of responder cells and the number of spots per well after subtraction of background. Responses were considered positive if a minimum of 15 spot-forming cells (SFC) were counted after the control had been subtracted and if the ratio of SFC between positive experiments and their respective control was larger than 3.

## Competing interests

The author(s) declare that they have no competing interests.

## Authors' contributions

NC carried out the DC culture, the T cell activation experiments and the ELISPOT assays. She also drafted the manuscript. PP carried out the T cell clone experiment and helped drafting the manuscript. ML participated in acquiring and analyzing flow cytometry data. GN kept cell lines into culture and performed the IFN-γ ELISA. DRVB and ML participated in coordination and analysis of flow cytometry. VFIVT participated in design and coordination of the study and critically revised the manuscript. ZNB reviewed and gave final approval of this version of the article to be submitted for publication.
